# *“You are helping from the heart not just from the head*”: a systematic review and qualitative evidence synthesis of the experiences of peer workers working with people experiencing homelessness and substance use

**DOI:** 10.1186/s12889-025-23006-6

**Published:** 2025-05-09

**Authors:** Hannah Carver, Joanna Astrid Miler, Jessica Greenhalgh, Bernie Pauly, Nicola Ring, Hazel Booth, Josh Dumbrell, Tessa Parkes

**Affiliations:** 1 Salvation Army Centre for Addiction Services and Research, Faculty of Social Sciences, University of Stirling, Stirling, Scotland FK9 4LA UK; 2 School of Applied Sciences, Edinburgh Napier University, Sighthill Campus, Edinburgh, Scotland EH11 4BN UK; 3 Canadian Institute for Substance Use Research, University of Victoria, Victoria, V8N 5M8 Canada; 4 School of Health and Social Care, Edinburgh Napier University, Sighthill Campus, Edinburgh, Scotland EH11 4BN UK; 5 School of Health Sciences, University of Dundee, Dundee, Scotland DD1 4HJ UK

**Keywords:** Substance use, Homelessness, Peer workers, Peer support, Qualitative, Qualitative evidence synthesis, Systematic review

## Abstract

**Background:**

Increasingly, substance use and homelessness services have peer workers, those with lived or living experience of substance use and homelessness, who provide support to those experiencing similar challenges. While research regarding the effectiveness of such peer workers in helping others achieve better outcomes is growing, little is known about their experiences in this role.

**Methods:**

A systematic review and qualitative evidence synthesis was conducted to better understand the experiences of peer workers who have lived/living experience of substance use and homelessness who are providing support to those experiencing similar challenges within substance use and homelessness settings. Nine electronic databases were searched for primary qualitative research published from 1990. Studies meeting the inclusion criteria were quality assessed using the Critical Appraisal Skills Programme checklist. Data from included studies were extracted, entered into NVivo, and analysed using a thematic synthesis approach.

**Results:**

Nine studies were identified, published from 2006 from three countries with 272 participants. Three themes were identified: peer workers’ reflections on the key components of their role; peer work as enabling individual growth and recovery; and destabilising challenges peer worker growth and recovery.. Peer workers described many essential qualities, and their lived experience was valued as a way of enabling deeper trust and empathy with the people they supported. Strong relationships with other peer workers were described as important. Many benefits to the peer workers were described, including positive life changes and increased responsibility. Challenges were also identified, with professional boundaries causing particular tensions.

**Conclusions:**

This qualitative evidence synthesis provides unique insight into the experiences of peer workers who are working at the intersection of homelessness and substance use. Their experiences highlight the real benefits that peer workers have, whilst working in challenging situations in often precarious contracts. Such insights can inform the employment of peer workers. Those employing peer workers should prioritise clear job descriptions encompassing specific peer qualities, training and education opportunities, and peer-to-peer, professional, and organisational support.

**Supplementary Information:**

The online version contains supplementary material available at 10.1186/s12889-025-23006-6.

## Background

Peer support refers to a process whereby individuals with lived experience of a particular phenomenon provide support to others by explicitly drawing on their experience of this situation to support others in similar circumstances [1]. The idea that peers can help others through specific life struggles (such as engaging with treatment or to access other supports) has long been established, especially within mental health settings where peers have been providing mutual support since the 1800 s [2].Support for peers in research, policy, and practiced as increased considerably since the 1970 s [3–6]. Internationally, peer support has since moved into other service areas including homelessness, criminal justice settings, substance use treatment, harm reduction, and physical health [7–10]. Individuals providing peer support frequently share a common experience of social and health issues, including, in the case of this review, homelessness and substance use challenges.

In relation to substance use specifically, peers can provide various types of support for those still experiencing problems with homelessness and substance use, at different points of their lives. This includes, harm reduction services, where the aim is to reduce the harm associated with substance use without any expectations around abstinence [11], or helping to navigate services when someone is entering recovery [9, 10]. In this paper, we use the term recovery to mean someone’s personal wellness journey which involves changes in their health and wellbeing and life purpose and can include harm reduction and abstinence goals [12]. Peers work extensively in harm reduction settings, providing safer use education and overdose prevention services [13–15]. Peer support can be informal, involving ad hoc support from one individual to another, and formal, with peers trained to offer support in a structured way, as well as paid or unpaid work. This paper focuses solely on this latter type of support, where peers with experiences of substance use and homelessness are in formal support roles working with people also experiencing homelessness and substance use challenges.

Globally, the value of peer workers is increasingly being recognised, as reflected in recommendations for peer support within guidelines for various health issues, and across multiple sectors [16, 17]. Peer worker visibility and importance is evident in the United Kingdom (UK), North and South America, Asia, and Europe [18–23]. In addition, there are examples of strategic policy support for peer support in substance use settings in Scotland [24], England [16], Australia [25], and Canada [26]. Moreover, recommendations are being made for an increase in formalised peer worker employment opportunities in the homelessness sector [18]. However, the types of work peer workers do, and their working conditions, vary considerably depending on the country and setting. For example, many Canadian peer workers work in overdose prevention settings [27, 28], whereas in the UK peer workers are generally found in abstinence-based recovery settings. As such, the roles of peer workers at the intersection of substance use and homelessness vary widely across the world. There is also the need to distinguish between peer workers with lived versus living experience, i.e., current homelessness and substance use versus previous experiences of these challenges. Some organisations only employ peer workers with lived experience (but not active substance use), whereas others also offer employment to those with living experience. Importantly, there is a growing body of evidence, especially in Canada, suggesting that peers with both types of experience can successfully lead the harm reduction movement in meaningful ways, contributing to the reduction of harms associated with substance use [28–32].

Despite the growing body of work on the impacts of involvement in peer support work in the context of mental health, HIV, hepatitis C and/or substance use support [33, 34], only a few studies have explored peer service provision within the context of homelessness [35], with positive impacts on clients, including quality of life, substance use and social support [36]. Within this field, there is little focus on the experiences of peers, as opposed to those using those services. Even less attention has been paid to those with lived experience of substance use and homelessness who are now working at this intersection within substance use and homelessness settings. It is likely that peer workers with experiences of both homelessness and substance use will face different types of challenges in their roles and have unique (and higher) support needs compared to those working in only one of these fields. The focus on both homelessness and substance use in this review is that this is an area in which the involvement of peer workers is growing, which could have potential benefit to some of the most marginalised members of society [36]. A previous ‘state of the art’ review synthesised the available evidence regarding peer support interventions that specifically address the intersection of homelessness and substance use [1]. Five key themes relating to the challenges faced by peer workers were identified including vulnerability, authenticity, boundaries, stigma, and lack of recognition [1]. While not its main focus, this was one of the first reviews to look at the potential impact of the role on the peer workers themselves. The qualitative evidence synthesis reported in this paper was inspired by Miler et al.’s (2020) findings [1], with the aim of developing an in depth understanding of peer workers’ experiences, which is missing from the evidence base. Miler et al.’s (2020) [1] state of the art review was very broad and focused on all literature on peer support models (including literature from the perspective of those receiving support, both quantitative and qualitative), whereas this review focuses solely on the experiences of peer workers in qualitative literature. This review aims to examine the perspectives of peer workers with experience of substance use and homelessness who are now working at this intersection, providing support to people facing both of these challenges, either in specific interventions or in service provision. Understanding peer workers’ experiences can provide essential information to organisations wishing to employ these workers in the future or improve the experiences of those currently employed, by understanding, and therefore avoiding, some of the key challenges.

## Methods

### Study design

Qualitative evidence synthesis refers to systematic reviews of qualitative research, bringing together findings across a range of studies to provide an in-depth understanding of a particular area of research [37]. Qualitative evidence syntheses go beyond simply summarising research findings to develop new knowledge in narrative form [38]. Flemming and Noyes (2021) note that there are more than 30 approaches to conducting qualitative evidence syntheses, with thematic synthesis, framework synthesis, and meta-ethnography being the most widely used and well-developed methods [38]. This qualitative evidence synthesis involved taking a thematic synthesis approach, using thematic analysis to generate new insights and understandings from a body of qualitative studies [39]. Thematic synthesis was chosen due to its ability to maintain links between the findings and conclusions of the primary studies, and its common use within qualitative evidence synthesis research [39]. Our thematic analysis addressed the research question: *how do individuals with lived/living experience of homelessness and substance use manage the process of providing support as peer workers to people experiencing these same challenges?*

After conducting a preliminary search to ensure the availability of a body of literature to be synthesised, we defined homelessness using the ETHOS definition which considers homelessness to cover a range of living situations including rooflessness, houselessness, insecure housing, and inadequate housing [40], with homelessness needing to be explicitly mentioned in the studies in order to be included. ‘Peer workers’ were defined as people with lived or living experience of homelessness and substance use who provide any form of formalised peer support to others at the intersection of homelessness and substance use within homelessness settings (i.e., those providing support/accommodation to those experiencing homelessness, such as hostels, temporary accommodation, drop-ins etc.). The study protocol was developed and registered with PROSPERO and subsequently updated to reflect a change in focus from their transitions into this role to their overall experiences, due to a lack of published literature on the former (CRD42022335800). The qualitative evidence synthesis was conducted according to the ‘enhancing transparency in reporting the synthesis of qualitative research’ (ENTREQ) statement [41] (Supplementary file 1).

### Search strategy

Systematic literature searching was conducted to identify all relevant primary qualitative studies relating to our research question. The SPIDER framework (Sample, Phenomenon of Interest, Design, Evaluation, Research type) [42] was used to identify appropriate literature search terms and create exclusion/inclusion criteria (Table 1). Nine electronic databases (CINAHL; Criminal Justice Abstracts; Health Source; MEDLINE; NIHR Journals; PsycINFO; Social Care Online; SocINDEX; and Web of Science) were searched, using key search terms in order to identify the relevant academic peer reviewed literature (Table 2). The searches were limited to qualitative or mixed-methods studies involving a substantial qualitative element where qualitative results were clearly described, reported, and published between 1990 and July 2022. While these nine databases were searched again in August 2023 using the same strategy, no new studies meeting the inclusion criteria were identified. To maximise capture of all potentially relevant data, no language limiters were applied. Reference lists of all included studies were also reviewed.

**Table 1:** Inclusion/exclusion criteria

Inclusion	Exclusion
Sample	
People with lived or living experience of homelessness AND substance use (including poly-substance use – i.e., concurrent use of various substances) in formal peer worker roles (paid or voluntary) Adults (aged 18 years and older, with no upper age limit)	Participants had not experienced substance use and homelessness or work in formal peer support worker roles Receipt of peer support reported but not experiences of support provision
Phenomenon of interest	
Formal peer worker roles delivered in homelessness and substance use settings, working with people experiencing substance use (drugs and/or alcohol) and homelessness Studies must examine the experiences of people with lived/living experience of substance use (drugs and/or alcohol) AND homelessness, who transitioned to become peer workers All types of formal peer support worker roles (including paid and voluntary roles e.g., a defined formal peer support worker role within the charitable sector)	Informal peer support experience only or formal peer worker role experience in settings other than homelessness and substance use Peer workers with lived/living experience with substance use or homelessness only
Design, Evaluation, Research type	
Experiences of peer workers from the perspective of the peer workers Any qualitative methodology; mixed-methods studies containing substantial qualitative components and sufficient depth of results Papers published between 1990-July 2022 (inclusive)/August 2023	Other experiences of peer workers e.g., evaluations of peer workers’ effectiveness and experiences of clients who receive peer support Quantitative research designs, not primary research e.g., editorials, other systematic reviews Papers published before 1990

**Table 2:** Example search strategy

PsychInfo
1. (Substance us* OR drug use* OP alcohol use* OR problem* substance use OR problem* alcohol use OR problem* drug use OR addiction OR substance dependenc* OR alcohol dependenc* OR drug dependenc* OR drug dependenc* treat* OR intervention OR recovery OR therap* service*)
2. (homeless* OR underhouse* or roofless*OR street involved OR rough sleep* OR unstable hous* OR housing instability OR precarious* hous* OR undomiciled OR houseless OR street person OR street people OR no fixed abode OR transient OR vagrant OR shelter OR unshelter OR* OR destitute)
3. 1 AND 2
4. (peer support worker* OR peer worker* OR peer mentor* OR peer specialist*OR peer nsvigstor* OR peer support* OR peer* OR support* OR buddy)
5. 3 AND 4
6. (Qualitative research OR qualitative study OR qualitative OR focus group OR interview* OR ethnograph* OR observation* or ETHNOGRAPH* or NARRATIVE or ACCOUNT or GROUNDED THEORY OR case study OR interpretative OR thematic analysis OR framework approach OR mixed method*)
7. 5 AND 6

### Selection criteria and quality appraisal

Full inclusion and exclusion criteria are provided in Table 1. Papers that specifically focused on youth and those concerning informal peer support arrangements, such as support to friends, were excluded. Because the focus of this review is peer work at the intersection of homelessness and substance use, only studies which explicitly mentioned both homelessness and substance use were included (in terms of both peer worker experiences and the setting in which they worked). This meant that a range of studies were excluded, such as those focusing on peer work in harm reduction if homelessness was not explicitly mentioned.

Initial searches and deduplication were performed by one reviewer (JM). Two reviewers performed screening by title and abstract (JM screened 100%, and HC screened 20% of the titles and abstracts, in parallel) using Rayyan. Any disagreements were resolved by a third reviewer (TP). Once potential included studies had been identified, full texts were screened against the inclusion criteria by one reviewer (JM). A wider team with different required expertise met, reviewed, and agreed on the included papers (JM, HC, TP, BP, HB, and NR). Any disagreements were resolved through full team discussion and consensus. No outreach to authors was conducted as this was not deemed to be required. The updated searches were performed by JG, with HC and JG reviewing all potential titles and abstracts. No new studies were identified. Reference details identified through the literature search were collated and managed using Rayyan. Literature searching and screening results were reported using PRISMA [43].

Studies meeting the inclusion criteria were quality assessed using the Critical Appraisal Skills Programme (CASP) qualitative research checklist [44] (see Supplementary file 2). Both HC and JM independently appraised each study and then discussed the results. Scores were then combined to create the final CASP table (Supplementary file 2). Quality appraisal allowed for the systematic consideration of study strengths and weaknesses [45]: it was not used to exclude studies [46].

### Data extraction and analysis

Study characteristics including setting, participant characteristics, and methods were entered into an Excel spreadsheet. Thematic synthesis was conducted inductively using the three-stage approach described by Thomas and Harden (2008): line-by-line coding of individual studies; creation of analytical themes; and generation of new constructs, explanations, or hypotheses [39]. Following a thematic synthesis approach [37], first-order (participant quotes) and second-order (author interpretations) data were extracted and entered into NVivo version 20. This approach allowed datasets to be examined separately to look for differences between first- and second-order data, to inform new insights into the studies. First- and second-order data were coded line-by-line to identify themes and concepts. JM and HB performed the data extraction and JM led on the analysis, with HC checking for accuracy. Any disagreements were discussed until consensus was reached, with regular team meetings used for reflection to allow team members to challenge analytical processes and interpretations. First- and second-order codes were then written up narratively to explore relationships between the studies. In the final stage, the narrative synthesis for the first- and second-order data were combined, providing an overall synthesis of the key thematic areas reported across all nine studies. A final synthesis was created, with descriptive quotes from the primary studies to illustrate key points. These quotes have been standardised (in terms of italics, ellipses etc. rather than changing words) and therefore may be different to how they are presented in the original studies.

## Results

**Fig. 1:**
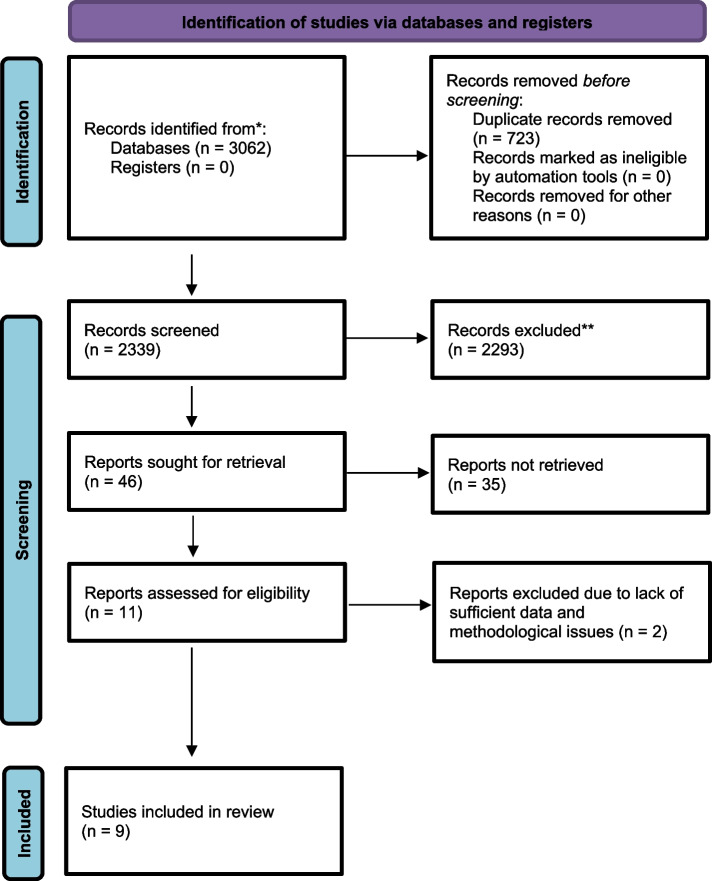
PRISMA flow diagram. PRISMA 2020 flow diagram for new systematic reviews which included searches of databases and registers only. *From:* Page MJ, McKenzie JE, Bossuyt PM, Boutron I, Hoffmann TC, Mulrow CD, et al. The PRISMA 2020 statement: an updated guideline for reporting systematic reviews. BMJ 2021;372:n71. https://doi.org/10.1136/bmj.n71

Nine studies were identified as meeting the inclusion criteria (see Fig. 1).

Six of the studies were conducted in the UK: five in England [34, 47–50] and one across Scotland and England [36]. Two studies were conducted in Canada [27, 51] and one in the United States (US) [52]. Studies were conducted between 2006 and 2022 with a total of 272 participants. Data were collected using individual interviews, focus groups, participatory case studies, and observations. Studies were of varying quality, with half viewed as high quality (scoring 9 +/10 on the CASP quality appraisal tool), and the other half deemed lower quality (scoring 4–7/10).

Peer workers were based in homelessness, substance use and/or healthcare settings, including outreach settings. Peer workers’ own substance use status varied across studies: in four studies peer workers reported current or past substance use [27] and in the other five studies they reported past use, implying abstinence [34, 36, 47, 49, 50]. Table 3 highlights the characteristics of the included studies in terms of settings and participant demographics.

**Table 3:** Characteristics of included studies (chronological order)

Authors	Country	Setting	Study aim	Participant information	Definition of peer support	Methods	Key findings of the study
Weeks et al. (2006) [52]	Connecticut, US	Outreach settings where people are using drugs including parks, soup kitchens, homeless shelters, abandoned buildings, and alleyways	To understand the impact of the Risk Avoidance Partnership (RAP) project in terms of the training provided to peer/public health advocates and their experiences of the intervention programme	Candidates who received the intake interview (*n* = 176) and initiated training programme (*n* = 130), who experienced drug use, who became peer health advocates. Majority were male (61.6%) and from diverse ethnic backgrounds (African American, Puerto Rican, non-Hispanic White)	Peer health advocates were actively using drugs who were trained to provide a structured, peer-led intervention to those using drugs. Received monetary and non-monetary compensations	Observations during in-office training sessions (n = 25) and partnered training sessions in the community (n = 66)	The peer health advocates reported a significant positive role change in themselves while conducting health advocacy work, and willingly and successfully carry the peer-led intervention into locations of high-risk drug activity. They successfully conducted full engagements, providing education, materials, and, less often, demonstration of proper use of the harm reduction materials with peers in a variety of settings, without project staff support
Croft et al. (2013) [47]	England, UK	Specialist outreach team focused on tuberculosis (TB) amongst those experiencing homelessness, drug and/or alcohol problems	To understand the motivation and personal impact of being a peer educator on people with experience of anti-tuberculosis treatment, homelessness, and addiction	Peer workers (*n* = 7) with current/recent experience of working as peer educators. Five males of diverse ethnic origin (Eastern European, British Black Caribbean, South Asian, Black Caribbean) and one white, UK born female	Peer workers with experience of homelessness, TB and drug/alcohol dependency. No other details provided regarding intervention or payments	Individual semi-structured interviews to understand the motivation and personal impact of being a peer educator Analysis: grounded theory	Participants reported that being a peer educator can help them make sense of past experiences and renew their sense of self, helping with their long-term recovery. The motivational themes identified highlight changes in self-perception that can occur as a result of being a peer, where what motivates initial volunteering is the transition from treatment, followed by the perception of new opportunities such as training or employment
MacLellan et al. (2017) [48]	England, UK	Randomised controlled trial (RCT) of a peer-delivered intervention for improving patient engagement with National Health Service (NHS) services amongst those with a hepatitis C diagnosis who experience homelessness and injecting drug use	To explore how peer advocates achieve their connections to facilitate wellbeing and health service engagement of their client	Peer advocates (*n* = 5) whose role was to engage with clients and support/advocate for them through the appointment process and generally within services. All male; ethnicity not reported	Peer advocates with experience of homelessness and substance use who were supporting people experiencing hepatitis-C, injecting drug use and homelessness. No other details provided regarding intervention or payments	Individual narrative interview to understand how peer advocates made and sustained relationships with clients Analysis: structural narrative analysis	Peer advocates all had different approaches to managing the client-health provider relationship, depending on their personality, personal identity, and life experience. Participants discussed the challenges and techniques of engaging with clients, with the health provider relationship of lesser concern. The setting of boundaries was an important component to minimise client dependency and protect their recovery process. Self-disclosure needs to be acknowledged as a key component of the therapeutic use of self
Barker et al. (2018) [49]	England, UK	Four homelessness third sector (not-for-profit) organisations	To understand the critical elements of intentional peer support on those who provide and/or experience this support	Current providers of intentional peer support (IPS) (*n* = 28). *(One service user recipient was also interviewed but their data were excluded from our analysis).* 80% of participants were male; ethnicity not reported	Peer workers providing IPS (specific roles in organisations filled by those with lived experience). Peer worker roles differed across the four organisations involved, none of whom were paid for their work	Individual semi-structured interviews to explore experiences of IPS Analysis: thematic analysis	Peers'persistence in developing unique experience‐based relationships, providing social support, role modelling recovery, and peers'motivations were perceived as important factors involved in peer support. Peers described benefitting from helping, such as, undergoing transformative identity developments that helped them to escape homelessness. Through the re-telling of their stories, they create meaning and restructure their autobiography, attributing experiences of homelessness as a catalyst for positive changes in their lives
Tookey et al. (2018) [51]	Ontario, Canada	Three community-based health centres with onsite specialist support from nearby hospital	To gain an understanding of the transition from client to support worker from the perspective of two individuals who were involved in the project	Community support workers (*n* = 2) employed to provide support to people living with hepatitis C. No gender or ethnicity information reported	Peer workers were those who had received treatment for hepatitis-C. Training offered to current or former clients and aimed to increase their capacity to work as support workers within the programme or in other organisations. Training programme involved 2-h sessions over 16 weeks. All paid an hourly wage, as well as holiday and sick pay	Participatory case study approach with two of the five workers to explore the transition from client to support worker Analysis: inductive approach	The transition from client to co-worker described as a gradual process and one that is supported by, and in turn helps to support, a number of other personal transitions. Prior experience, changes in substance use practices, shifts in relationships with community members and friends, supportive organisational and structural factors, and role transition were highlighted as facilitators and challenges
Pauly et al. (2021) [27]	British Columbia, Canada	Two substance use/homelessness organisations	To identify, implement and evaluate support for peer workers in overdose response environments,	Peer workers (*n* = 31) working in overdose response environments. 55% male and 45% female; ethnicity not reported	Peer (‘experiential’) workers had past or present drug use experience who are using that experience in their professional work. Work includes distribution of harm reduction supplies, peer witnessing of drug use, referrals to other agencies, advocacy, outreach work and overdose response. No details about training or payment	Focus groups (n = 8) to explore peer workers’ roles, positive aspects of the role, challenges, and support needs Analysis: interpretative description	Peer workers described a range of motivators for their role: a sense of purpose from helping others; being an inspiration for others; and a sense of belonging
Surey et al. (2021) [34]	England, UK	Homelessness organisation	To explore the experience of transitions from ‘street’ to ‘institution’ of those working as peer workers with those experiencing hepatitis C	Advanced peer support workers (*n* = 5) whose role involved active case finding, relevant healthcare testing and referral to other services. *(Service users and service providers also interviewed but their data were excluded from our analysis).* All male; ethnicity not reported	Peer support workers with lived experience of homelessness, drug use and hepatitis-C. Trained in blood borne virus testing, liver fibrosis assessment, referrals and support. All paid roles and equal members of team	Individual semi-structured interviews, conducted by one of the peer workers Analysis: thematic analysis	The findings explored transition to integration; retaining ‘peerness’; and practising critical resilience. The findings revealed a supportive programme structure as participants make the move between groups, supporting their employability and development of critical resilience
Annand et al. (2022) [50]	England, UK	Homeless health peer advocacy service run by third sector (not-for-profit) organisation	To understand the impact of Homeless Health Peer Advocacy on peer advocates	Current and former peer advocates (*n* = 14). *(Staff members (n* = *2) and stakeholders (n* = *3) also interviewed but their data were excluded from our analysis).* Peer advocates were 60% female, 40% male; from diverse ethnic groups (Black British/Black African, mixed ethnicity, white)	Peer advocates had experience of homelessness who were trained to provide support to help people access healthcare, such as accompanying people to appointments and providing support before, during and after appointments. All unpaid volunteers	Individual semi-structured in-depth interviews (two peer advocates interviewed twice to explore experiences in more depth) to explore peers’ experiences as a peer advocate Analysis: grounded and abductive approach	The impacts of being a peer advocate revolve around progression capitals, which peers appear to have developed further through their engagement with the project. These include the social capital generated by the relationships afforded them upon joining the team; cultural capital gained via the organisation’s approbation of lived experience, and provision of health advocate status; human capital via extensive training and clinical support; and physical capital via a bursary scheme
Parkes et al. (2022)[36]	Scotland and England, UK	Third sector (not-for-profit) homelessness services, three outreach services and three residential services	To understand the experience of those working as Peer Navigators	Peer Navigators (*n* = 4) who provided emotional and practical support to clients. *(Staff (n* = *12) and clients (n* = *24/n* = *10 also interviewed but their data were excluded from our analysis).* No gender or ethnicity information reported	Peer Navigators had lived experience of homelessness and/or substance use and received extensive training on wide range of areas. All paid roles	Individual semi-structured interviews at three or four time points and reflective diaries, to explore peer navigators’ experiences in their role Analysis: framework analysis	The peer navigators were employed in demanding professional roles, providing unique support to their clients, as well as making complex decisions, holding responsibilities for clients’ personalised budgets, and case management, as well as performing a range of tasks. The peer navigators reported a range of benefits and challenges with their role

As the included studies focused on peer workers’ experiences in their role at the intersection between homelessness and substance use, the synthesised findings reflect these experiences and are reported as three over-arching themes: the key components of the peer worker role; benefits of being a peer worker; and challenges of being a peer worker. Each theme and their supporting sub-themes are reported below. Table 4 provides an overview of the themes and sub-themes, and Table 5 provides detail of which paper is reflected in each theme and sub-themes.

**Table 4:** Themes and sub-themes

Theme	Sub-themes
Peer workers’ reflections on the key components of their role	What qualities are required to be a peer worker? Motivations for becoming a peer worker Shared experiences as a way of building connections and trust Capacity building
Peer work as enabling individual growth and recovery	Peer work as being emotionally beneficial Positive life changes as a result of peer work Peer work as providing opportunities for developing a sense of responsibility
Destabilising challenges to peer worker growth and recovery	The impact of own and others’ substance use on growth and recovery Emotional difficulties associated with the role The difficulties of setting boundaries The impact of clients’ difficult lives, circumstances, and behaviours on peer workers Tensions between peer workers and other professionals

**Table 5:** Theme and sub-theme breakdown by study

	**Annand et al. (2022)**	**Barker et al. (2018)**	**Croft et al. (2013)**	**MacLellan et al. (2017)**	**Parkes et al. (2022)**	**Pauly et al. (2021)**	**Surey et al. (2021)**	**Tookey et al. (2018)**	**Weeks et al. (2006)**
Themes	**Sub-themes**								
Peer worker reflections on the key components of their role	What qualities are required to be a peer worker? Motivations for becoming a peer worker Shared experiences as a way of developing connections and trust Capacity building	What qualities are required to be a peer worker? Motivations for becoming a peer worker Shared experiences as a way of developing connections and trust Capacity building	What qualities are required to be a peer worker? Motivations for becoming a peer worker Shared experiences as a way of developing connections and trust Capacity building	What qualities are required to be a peer worker? Shared experiences as a way of developing connections and trust Capacity building	What qualities are required to be a peer worker? Motivations for becoming a peer worker Shared experiences as a way of developing connections and trust Capacity building	Motivations for becoming a peer worker Shared experiences as a way of developing connections and trust Capacity building	What qualities are required to be a peer worker? Motivations for becoming a peer worker Shared experiences as a way of developing connections and trust Capacity building	What qualities are required to be a peer worker? Motivations for becoming a peer worker Shared experiences as a way of developing connections and trust Capacity building	What qualities are required to be a peer worker? Motivations for becoming a peer worker Shared experiences as a way of developing connections and trust Capacity building
Peer work as enabling individual growth and recovery	Peer work as emotionally beneficial Positive life changes as a result of being a peer worker Peer work as providing opportunities for developing a sense of responsibility	Peer work as emotionally beneficial Positive life changes as a result of being a peer worker Peer work as providing opportunities for developing a sense of responsibility	Peer work as emotionally beneficial Positive life changes as a result of being a peer worker Peer work as providing opportunities for developing a sense of responsibility	Peer work as emotionally beneficial Benefits of peer support to clients	Peer work as emotionally beneficial Positive life changes as a result of being a peer worker	Peer work as emotionally beneficial Positive life changes as a result of being a peer worker Peer work as providing opportunities for developing a sense of responsibility	Peer work as emotionally beneficial Positive life changes as a result of being a peer worker Peer work as providing opportunities for developing a sense of responsibility	Peer work as emotionally beneficial Positive life changes as a result of being a peer worker Peer work as providing opportunities for developing a sense of responsibility	Peer work as emotionally beneficial Positive life changes as a result of being a peer worker Peer work as providing opportunities for developing a sense of responsibility
Destabilising challenges to peer worker growth and recovery	The impact of own and others’ substance use on growth and recovery The difficulties of setting boundaries Tensions between peer workers and other professionals	The impact of own and others’ substance use on growth and recovery Emotional difficulties associated with the role The difficulties of setting boundaries The impact of clients’ difficult lives, circumstances, and behaviours on peer workers Tensions between peer workers and other professionals	The impact of own and others’ substance use on growth and recovery Emotional difficulties associated with the role The difficulties of setting boundaries The impact of clients’ difficult lives, circumstances, and behaviours on peer workers Tensions between peer workers and other professionals	Emotional difficulties associated with the role The difficulties of setting boundaries The impact of clients’ difficult lives, circumstances, and behaviours on peer workers Tensions between peer workers and other professionals	The impact of own and others’ substance use on growth and recovery Emotional difficulties associated with the role The difficulties of setting boundaries The impact of clients’ difficult lives, circumstances, and behaviours on peer workers Tensions between peer workers and other professionals	The impact of own and others’ substance use on growth and recovery Emotional difficulties associated with the role Tensions between peer workers and other professionals	The impact of own and others’ substance use on growth and recovery The difficulties of setting boundaries Tensions between peer workers and other professionals	Emotional difficulties associated with the role The difficulties of setting boundaries The impact of clients’ difficult lives, circumstances, and behaviours on peer workers Tensions between peer workers and other professionals	The impact of own and others’ substance use on growth and recovery Emotional difficulties associated with the role The difficulties of setting boundaries The impact of clients’ difficult lives, circumstances, and behaviours on peer workers Tensions between peer workers and other professionals

### Theme 1: Peer worker reflections on the key components of their role

In all nine studies, peer workers reflected on the key components of their role. These key components are described in four sub-themes: the motivations for becoming a peer worker; the qualities required to be a peer worker; shared experiences as a way of developing connections and trust; and capacity building.

#### Motivations for becoming a peer worker

Participants in eight studies talked about their motivations for becoming, and continuing to work as, peer workers [27, 34, 36, 47, 49–52]. Peer workers described how they were driven by an “*overwhelming*” [49] desire to help others [51]:

*… you develop sympathy for the people that you are helping, and you see that you are helping from the heart not just from the head.* [[47]; p.S38]

Some discussed how their motivation was specifically to provide support to those individuals who had, at that point in time, been described by other colleagues as hard to engage with [36]. Others talked about how saving lives and reversing an overdose had given their work meaning and served as a motivator:

*I really like that in a way, when you reverse an overdose for somebody, you’ve given them another chance at life. It’s a pretty profound experience.* [[27]; para.25]

Wanting to give something back to the community (or the organisation that provided support to them in the past), and making up for previous perceived wrongdoings, were highlighted by peer workers in three studies [34, 49, 51]. Peer workers talked about their role as providing opportunities for change and to give something back to society:

*It feels like a bit of karma, a bit of balancing the scales if you like because when I was 21 I was a menace to society according to a Crown Court Judge and now I am not a menace to society.* [[34]; para.30]

While peer workers highlighted that some of their early motivators for becoming involved with peer support as a volunteer or employee were monetary (such as getting paid or getting other cost benefits, including food) [51], with time personal motivations commonly changed. For example, one of the motivations reported as time progressed was to aid the individual’s own recovery and learn more about themselves via self-awareness [52]. Some highlighted their feeling that peer support roles had become “*part of [their] nature*” [47]. Yet, some peers also discussed the nuances of paid versus voluntary roles, with some expressing the view that the role should be unpaid in order to reflect true altruistic and genuine motivation to help others [56]:

*It’s not something I get paid for, it’s something that I really believe in and… I’m not too sure I could… do it* [as] *a paid role.* [[50]; para.56]

On the whole, the motivation to do peer work was largely compassionate, with a genuine desire to help, wanting to give back to society and save lives. This is of particular importance, as peer work continuous to be largely inadequately remunerated (e.g. [29, 49]) and thus poses the question whether the compassionate motivation to undertake such role precipitates or reinforces the low wages or lack of payment.

#### What qualities are required to be a peer worker?

The qualities required to become a peer worker were discussed in eight studies [34, 36, 47–52]. Being able to work using their own initiative [48], working with their intuition, and displaying tenacity in not giving up on people, were all seen by peer workers themselves as crucially important:

*I would go away, but they would still be in my mind. In my mind I’m already preplanning, I’m coming back next week, I won’t give up.* [[49]; p.219]

Also discussed was the need to provide person-centred [48, 49] and holistic care to those they support, which was often far wider than just substance use:

*It was difficult because* [name of staff] *wanted me to just be working with them around the drugs and alcohol. But obviously, when people are coming into me and you are doing a whole holistic thing around all the trauma they have suffered, you are not just sat there talking about drugs and alcohol, you are talking about sexual abuse, about them working on the streets, about all the different things.* [[36]; p.73]

Showing a keen interest in the lives of the people they support, leadership skills, self-belief, and confidence were also seen as important in being successful as a peer worker [34, 48, 51]. Authors in some studies found that the peers who were no longer experiencing homelessness and substance use challenges in their own lives viewed themselves as more successful in their peer worker roles [34, 51].

#### Shared experiences as a way of developing connections and trust

The lived experiences of peer workers and the experiences they shared with the people they supported were central to all nine studies. Shared experiences of homelessness and substance use were seen as instrumental to their roles as they enabled peers to develop deeper levels of empathy:

*Lived experience acted as a conduit to an expression of empathy, respect and unconditional positive regard.* [[36]; p.70]

The peer workers reported having deeper connections with the people they supported compared to colleagues without such lived experience. This helped peers to identify the challenges people were likely facing but perhaps not talking about:

*Like when you’re homeless you pick up very well on certain things like vibes, energies, intentions, lies, you pick up very well on these things because more time you’re on the receiving end of those things.* [51; p.219]

The dual capacity of peer workers to operate as professionals while also drawing effectively on their personal experiences of substance use and homelessness was discussed in five studies [27, 34, 36, 48, 49]. The peer workers often described themselves a ‘bridge’ or translator between the people they were supporting and services, because of their unique position of having a shared membership with these two groups. These shared experiences were seen as a positive enabler and unique attribute of the role:

[Shared experiences] *make people that generally would not associate, associate.* [[27]; para.34]

Being a role model was discussed in three studies [27, 36, 49]. This was described in these studies as ways of helping to destigmatise the challenges that people were experiencing around substance use and homelessness. While peer workers also reported gaining respect from their peers for being in such a role, at times they felt uncomfortable with their peer worker status:

*Some participants, however, expressed their discomfort at being perceived as a role model, as they did not want to seem “different” or “better” than their clients.* [[51]; p.223]

This highlights an area of tension for peers in relation to shared experiences.

Related to their shared experiences, peer workers in seven studies [27, 34, 36, 47–50] described their strong ability to develop trust with the people they supported, which was deemed a fundamental aspect of developing positive relationships. Peers discussed talking about their own experiences to build trust with the people to whom they provided support [34, 36, 48, 49]. Disclosure of their own lived and living experience of substance use and homelessness was key to facilitating these relationships:

*So, I spoke with him, calmed him down, reassured him and told him everything about what I had been through and everything. He then realised that hang on this guy has been exactly the same as me and it sort of changed him. And it was just that thing, “Thank you, I’ll go and think about what you said”.* [[48]; para.44]

Many authors noted the importance of having boundaries on what and how much information is shared as a way of ensuring trust amongst the people with whom the peer workers were supporting. Trust as a theme was also discussed in relation to its importance in the relationship between peer workers and other professionals [27, 48]. In particular, acceptance from other workers to trust the peer workers to “*act as a bridge*” [48] for the people they supported when accessing services was seen as essential. Both peers and other professionals were striving to reach the same outcomes for these individuals:

*The ability of the PA* [peer advocate] *to gain trust and acceptance of healthcare providers to act as a bridge to the client’s successful engagement was seen through an emphasis on shared group membership. This eased the relationship between the PA and health provider in some cases, as they were regarded as working towards the same goal.* [[48]; para.29]

*The bridge is based on trust and compassion… the ability to establish trust when others may be unable to do so.* [[27]; para.21]

Furthermore, issues with developing trust with the people they supported were discussed in two studies [34, 36]. Some peer workers talked about the struggles these individuals had with being open and honest with them which impacted their ability to develop trust with them. Peer workers in these studies identified that trust with those they were supporting cannot be established overnight; time is required to establish a trusting relationship [36].

#### Capacity building

In all nine studies, peer workers described two important factors that enabled them to build capacity and grow in their roles: support from others and skills development and training.

The support provided to peer workers to enable them to work effectively was discussed in all nine studies [27, 34, 36, 47–52].

Peers discussed the connections they had with other peer workers and the importance of such relationships. They described providing support for one another [36], as well as having a common goal, despite often having different life experiences:

*There* [are] *some people from different walks of life here. Even though we’re all kind of the same in one area, we’re all very different in others. There* [are] *so many differences, yet there’s a commonality. We bond over the same things.* [[27]; para.42]

Peers also discussed the support received from other professionals working in the services, which included instances of mentoring [36], emotional support for the peers themselves [49], and clinical supervision [36, 49–51]. Such support from others was described by the peer workers as enabling them to make positive changes in their own lives [47], as well as facilitating positive outcomes for the people they supported [49, 50]. Often, support for peer workers aided them in their transition from a receiver of support to deliverer:

*If we have issues, such as triggering, we can bring it there* [clinical supervision]… *we can have a one-to-one with the clinical supervisor as a one off and say, “Look, this is really bothering me, can we meet and talk?”* [[52]; para.35]

Feeling like part of the wider team, being treated as an equal and experiencing a sense of belonging, were also apparent in some of the studies [36, 47, 49]. The feeling of being part of the team demonstrated in these quotes seems to reflect strategies within organisations/projects to flatten the hierarchy, and for those in positions of authority and power to set the tone and culture of the team:

*It was nice that everyone was listening to everyone’s ideas no matter if you had been in research for 20 years or if you were brand new to the study. It’s like… everyone took on everyone else’s ideas, there was no hierarchy*. [[36]; p.78]

*You were treated as an equal. There was no condescending. And you would get that with different organisations.* [[47]; p.S38]

Peer workers in six studies discussed how the role gave them opportunities for professional and career development [34, 36, 47, 49–51]. This included being able to attend training and learn transferable skills [34, 36], receiving financial help from the organisation for educational purposes [50], and being able to grow as a person and learn from their experiences [49]:

*If I need anything, anything regarding education they will fund you for that.* [[50]; para.16]

Peer workers in three studies [34, 36, 50] noted that their role was more flexible in terms of role expectations and responsibilities than other professionals, which was viewed as beneficial:

*The health professional has a narrowness of purpose which you need to have to do the task whereas I don’t have to burden myself with that. I’m quite free.* [[34]; para.29]

This level of freedom and flexibility allowed peers to “*work beyond the service they were based in*” [36], for example, accompanying clients to appointments, supporting them with purchasing household items, and meeting informally outside of the service environment.

Training was discussed by peer workers in four studies [36, 48–50], mostly in a positive light, with peer workers talking about gaining transferable skills [36] which they hoped would help long term with their employability prospects [50]:

*If HHPAs* [homeless health peer advocates] *have their eye out and it’s something that they would like to do, they can… When I am ready to go back into the employment field, I think I’ll have a lot of things on my CV that’ll be looked upon favourably.* [[50]; para.27]

However, some peer workers thought more training was needed for them to be able to conduct their work more successfully. Issues regarding training, education, and career development were discussed in six studies [34, 36, 48–50]. Peer workers also described feeling underqualified to deal with all of the needs that clients had [48], losing confidence in oneself with regards to training and completing job applications [51], and having no education resulting in feelings that a career is unattainable:

*Some of us peers have no or very little education and a career seems a million miles away.* [[34]; para.17]

### Theme 2: Peer work as enabling individual growth and recovery

All nine studies highlighted factors relating to the benefits of being a peer worker which enabled individuals to grow and develop. These benefits are described across three sub-themes: peer work as being emotionally beneficial; positive life changes as a result of peer work; and peer work as providing opportunities for developing a sense of responsibility.

#### Peer work as being emotionally beneficial

All nine studies reported emotional benefits for the peer workers including: feeling good about doing something positive [52]; being an inspiration to others [27]; and being able to reappraise past experiences and feel like the (often painful) past was not “*for nothing*” [[27]; para.30]:

*You know there was a part of my life that for years and years I was very embarrassed about. Quite ashamed, you know… that I had and I wasted so much of my life. And coming here, I realised well, actually it's not a waste, its qualifications... It's when you can stand up and say, “well that's my experience”... That is something you cannot be taught... I was out there and instead of looking at it like a waste of time and as a victim, actually what I was doing was gaining my qualifications.* [[49]; p.219]

The peer worker role was regarded as meaningful, providing a sense of agency [48, 50], and enabling peer workers to feel proud of themselves and their role:

*I’m not sure what the word is, but I do feel proud of it.* [[34]; para.31]

Peer workers experienced a range of positive emotional interactions through their involvement in supporting the delivery of interventions. Participants talked about feeling “*special*” [36; p.77], and viewed themselves as an “*important part of the puzzle*” [[34]; para.31] in aiding others in their own recovery journey.

#### Positive life changes as a result of peer work

Eight studies [27, 34, 36, 47, 49–52] reported positive life changes, including substance use, such as being able to focus on something other than drugs:

*And you are focusing on something other than the streets, or on getting high and just copping* [obtaining] *drugs.* [[52]; para.34]

Learning new skills around, and therefore practicing, safer drug use (amongst those who continued to use drugs) as well as feeling “*solid*” in their own recovery [[36]; p.78] due to the work they were doing, was also discussed:

*There’s much better ways of doing… using drugs, so that’s how I practice. Drug, set, setting.* [[51]; para.29]

Additional positive changes reported included learning how to ‘tolerate’ the drug use of others and empathise with different individuals’ circumstances. Peers developed their own skills to help manage internal battles whereby they wanted clients to reach the same level of ‘recovery’ they were currently at, at the same time as understanding that individual will face a variety of personal obstacles:

*Tolerance, tolerance… when you go back into that community and there’s that atmosphere it makes you realise how hard it is and you develop sympathy for people that you are helping.* [[47]; p.S38]

The positive life changes reported by the peers show their own greater levels of stability, improved recovery outcomes, and increased quality of life, helping them to progress as individuals.

#### Peer work as providing opportunities for developing a sense of responsibility

A sense of responsibility in their own lives and their work was reported as another benefit for peer workers in seven studies [27, 34, 47, 49–52]. This level of responsibility came from feelings that others in the community looked up to the peers, which helped to keep the peer workers motivated in their roles:

*I really realized that, yeah, I know a lot of the people coming into the program and a lot of people were looking up to me at the time because I had helped implement all these different programs* [as part of the patient advisory board] *and stuff so I figured, well hey, might as well just keep going with it and see what happens.* [[51]; para.26]

Some participants talked about hoping to turn the role into a career by enabling them to progress in their own lives:

*As far as this program, it's brought me to the forefront,'cause being involved in this and doing outreach work, it's given me some sense of responsibility. You know when you out there in addiction, it's easy to say, “Oh I'm gonna do this, I'm gonna do that” and then push it to the side. But then when people ask you things and they reaching out and I say things, I try to make it mean something.* [[52]; para.36]

Being responsible for others, and being trusted with that level of responsibility, helped the peers to further develop skills benefitting their own progression, acting as a role model, “*to derive both pride and happiness from their work”* [[27]; para.37].

### Theme 3: Destabilising challenges to peer worker growth and recovery

In all nine studies, challenges of the peer worker role which could impact individual’s growth and recovery were described. These challenges are described across five sub-themes: the impact of own and others’ substance use on growth and recovery; emotional difficulties associated with the role; the difficulties of setting boundaries; the impact of clients’ difficult lives, circumstances, and behaviours on peer workers; and tensions between peer workers and other professionals.

#### The impact of own and others’ substance use on growth and recovery

Seven of the studies identified substance use issues as challenges for peer workers [27, 34, 36, 47, 49, 50, 52]. Some peer workers, who were no longer using substances, discussed their initial personal challenges with the harm reduction model and discomfort trying to reconcile it with their ideas of recovery:

*From the job I was doing before, treatment, very in line with my recovery model… This is going to be very different. It’s going to be very different doing harm reduction.* [[36]; p.79]

Others talked about the heartbreak of losing friends and family members to substance use, and the impact that that had on them as a person and their ability to do their job:

*I lost a couple of my best friends in the last couple of years and it’s just been really friggin’ hard.* [[27]; para.29]

Peers in several studies discussed the importance of having clear relapse policies for their roles, where one of the conditions of being a peer worker was being drug-free:

*A lot of people won’t disclose the fact that they have a substance misuse background and the difficulty is owning that stuff. Having a clear relapse policy as part of someone’s contract would help. And highlighting and celebrating the fact that people are in recovery, rather than having secrecy about it.* [[34]; para.26]

In addition, peer workers in two of the studies [34, 50] talked about the challenges relating to triggers, although these were conceptualised more broadly than just triggers regarding substance use and included wider issues such as mental health challenges [50]:

*Some people… they’ve got mental health or… sometimes it just ends up too much… it’s not easy if you’ve got lived experience… it can be triggering… it’s just at what stage people are in their lives.* [[50]; para.40]

The peer workers who talked about triggers in their jobs had quite different opinions regarding it. Some had talked about the role having the potential to trigger negative emotions and talked about how having lived experience was hard, whereas others seemed to think that “*the trigger model*” was an excuse, and that peer workers should “*just get on with it*” [[34]; para.14].

#### Emotional difficulties associated with the role

Peer workers discussed the emotionally challenging aspects of their role in seven studies [27, 36, 47–49, 51, 52]. These related to difficulties dealing with uncertainty and the role being an “*emotional roller coaster*” [[27]; para.30].

The need to allow time for processing feelings and to be able to heal was identified as another potential challenge:

*I think it takes time for people to start to feel comfortable in their roles and for people to take it on.* [51; para.33]

Seven studies [27, 36, 48–52] identified additional issues relating to these emotionally demanding roles. These included peer workers’ ongoing vulnerability [48]; current homelessness [52]; discomfort in being a role model [49]; and the discomfort they faced when having to perform outreach in certain neighbourhoods [48]. Three studies discussed feelings of stress and worry [27, 36, 52], as additional challenges:

*Along with positive experiences, benefits and sense of purpose experiential workers derived from their work, several commented that they work in very stressful and emotionally taxing environments.* [[27]; para.30]

#### The difficulties of setting boundaries

Eight studies identified challenges around peers setting boundaries within their role [34, 36, 47–52]. These included uncertainties regarding whether the relationship with the people they supported should be a friendship or not [48] as well as being available out of hours, or using their own money to purchase food, cigarettes or alcohol for those they supported [49]. At the same time, some peer workers discussed that crossing boundaries was sometimes necessary or unavoidable in order to keep people safe and provide extra support [49] as well as to build and maintain relationships:

*I do answer their phone calls because I feel that if they are calling me in this moment then I am important to them and I’m not supposed to but if they’ve got no-one else to call and they are calling me then it must be important, and I think it helps with that relationship that I answer, I’m not just another person that is ignoring.* [[34]; para.29]

Some study participants added that they experienced difficulties maintaining boundaries when living in the same neighbourhoods as the individuals they supported [51]. There was therefore an internal conflict faced concerned with either crossing boundaries to help someone or feeling the need to reinforce boundaries for the peer’s own personal protection and wellbeing:

*That's been one of the trials and tribulations of this job - is knowing your boundaries, because even when I’m done work, you know, and I'm used to going out partying with these guys. I can't do that as much anymore cause they kind of look up at me as a role model up here or whatever. You want to call it right so I can't be going out there and getting in fights and stuff. I have to be able to walk away from things and even though it's not, I'm not at work I still have to practice that.* [[51]; para.30]

In addition, the fluidity of the role and having no start and end points, with no “*road map*” for guidance, was a challenge faced:

*… and this work doesn’t have an end and it doesn’t really have a clear start either, you know what I mean? It’s fluid… There’s no road map for it.* [[51]; para.36]

This fluidity was described as causing difficulties for peer workers to maintain boundaries with the people to whom they were providing support. While peer workers highlighted the importance of maintaining such boundaries, they also felt that some clients could become too dependent on them:

*I do explain to them, like you know, even though I would love to help you I just can’t, I’m not clued up to do that... you do need a barrier there for your own sanity because it is frustrating.* [[48]; para.21]

Peers reported struggling to navigate such “*professional boundaries*” [[49]; p.223] while still being supportive of people’s wants and needs, adding that they often felt the need to self-disclose their own past experiences, but this needed to be balanced appropriately:

*You can feel the tension and then you can, then you think maybe I’ll better just say “oh yeah I used to be a drug addict, but I had a little help I got through it, you know it is possible”, something like that. Just saying that you open your hand, your cards up. It makes them trust you straight away a bit more. So you’ve got to share a bit but not too much.* [[48]; para.24]

In particular, there is a tension described here between being a peer and being a professional. On the one hand, peer workers described the need for authentic relationships with the people they supported through shared experiences and trust. On the other, there was the need for clear, professional boundaries, in terms of what information they could share and the support they could provide, in order to protect peer workers’ own wellbeing. Importantly, ‘consequences’ for crossing boundaries were discussed in two studies [48, 51], without any mention of what such consequences may be for either the peer workers or the people they are supporting.

#### The impact of clients’ difficult lives, circumstances and behaviours on peer workers

Challenges arising from working with the people the peer workers supported were identified in six studies [36, 47–49, 51, 52]. These related to having closer relationships with some people compared to others [36], and difficulties respecting individuals’ choices [48]. This included finding it hard to just ‘be’ with individuals who are acutely suffering without moving into ‘doing’ with or for them, as this participant eloquently describes:

*It’s a balance… just sitting around it sometimes is the hardest, most intense aspect of the job. Just sitting with someone who is obviously suffering quite a bit and just going “do you know what, I will sit with you while you feel like shit”.* [[36]; p.71]

Perceived difficulties relating to peoples’ behaviour and emotions were discussed in detail by peer workers in five studies [34, 36, 48, 49, 51]. These included feelings of frustration [50], people being viewed as volatile and, at times, unreliable [36], as well as having to deal with anger directed at them [49]. Challenges were also encountered because the people they supported were often very mistrustful of wider health professionals and services, which made it more difficult to persuade them to engage with them [34]. Indeed, there was a sense that peer workers had to mentally prepare themselves when experiencing such challenging behaviours, whilst also being mindful of the potential reasons for such behaviours, including wider systemic or structural problems, such as social inequalities and marginalisation:

*Sometimes they become even abusive, challenging behaviour, so if we just really, withdraw immediately because of that sort of abuse or behaviour or whatever, then definitely that person is not going to get the help. So that I expect, I expect and I have to be mentally ready you know not um fail because of that. Because I need to support that individual. So the first step is to come back. You know that's, that's really important. So that person today, is not angry at me, but is angry at something that is not related to me*. [[49]; p.222]

Finally, additional challenges highlighted in four studies [36, 48, 51, 52] included peer worker concerns regarding a lack of change in individual’s substance use and the consequent need to adjust expectations regarding outcomes, moving from a focus on abstinence to one of harm reduction. In addition, peer workers also described the difficulties of not being able to provide support to every person presenting at a service [36].

#### Tensions between peer workers and other professionals

All nine studies identified challenges arising from working collaboratively with other professionals and/or services. These related to differences in outlook/approach between peer and non-peer staff [34, 36, 49], challenges with some staff having difficulty accepting confidentiality between peer workers and the people they supported [36, 48], being treated differently to other staff [36, 49], and experiencing negativity from other staff, both colleagues internal to their own organisations and external agencies [36]. The barriers and constraints for peer workers, as well as being treated differently by other professionals, were highlighted strongly in the study by Parkes et al. (2022):

*My hands were constantly tied… every tiny thing that I wanted to do with someone, I’d have to run it by, like, three or four different people. And it’s like no one else had to do that. It just completely slowed everything down and made me feel like, you know, I wasn’t able to help anyone really because someone was going to come down on me about it… I got called into the office to explain why I’d questioned a certain member of staff. I am trying to say, ‘why am I not allowed to question another member of staff? I am not questioning the person to have an argument. I am questioning what they are actually saying and disagreeing with one of their comments about how we should maybe be doing things’.* [[36]; p.76]

Relatedly, hierarchy and power structures that negatively impacted on relationships between peers and other professionals, and the related impact on peer workers’ self-confidence, was discussed in five studies [34, 36]:

*… because you are surrounded by nurses all day and really smart people, it can be quite intimidating.* [[34]; para.20]

In one study, tensions reportedly arose between peers and other professionals due to differences in approach, for example in what was deemed as ‘professionalism’ by other workers and expectations to conform to professional norms. The peer worker in Parkes’ et al. (2022) study talked about their experience of having a different role to others and the comments they received from other staff:

*It can be a bit like ridicule sometimes, like “oh, are you going out for coffee with so and so again? Are you off to take him something to eat again, or are you buying him this again?” Do you know what I mean? Whereas they’re not, like, saying how I got three/four homeless people housing after three days.* [[36]; p.75].

Underpinning such misunderstandings, or the invisibility of the achievements of peer workers as highlighted in the quote above, seemed to be a lack of understanding from other professionals regarding the remit of the peer worker role which led to clashes [36]. Peer workers reported that some staff seemed to find it hard to witness the ease at which peer workers established connections with the people they supported [36], being personally and professionally threatened by this, as well as peers themselves experiencing feelings of intimidation when working with experienced medical professionals.

## Discussion

This paper presents the findings of the first qualitative evidence synthesis to explore the experiences of peer workers with lived/living experience of substance use and homelessness who are providing support to individuals with similar experiences in formal peer support roles within substance use and homelessness settings. The findings of nine studies were synthesised and reported as three main themes: peer workers’ reflections on the key components of their role; peer work as enabling individual growth and recovery; and destabilising challenges peer worker growth and recovery, and a range of sub-themes. This is the first qualitative evidence synthesis to explore the experiences of peer workers with experience of homelessness and substance use who are now working at this intersection, as they navigate their roles. As noted previously, recognition of the benefits of peer support has increased in recent years, leading to greater involvement of peers providing formal support to individuals experiencing challenges around homelessness and substance use as part of services in both substance use and homelessness services. This qualitative evidence synthesis specifically focuses on the perspectives of peer workers themselves at the intersection of homelessness and substance use to provide a synthesis of a key area of peer work that has received little attention. While our original intention was to synthesise evidence related to role transitions, we did not find studies that focused specifically on transitioning to the role, and support for such role transitions. However, in synthesising the evidence related to peers’ roles, we gleaned substantive insights into strategies for enhancing the support to those transitioning into peer roles. We now focus on the insights from this synthesis for supporting peer work.

The peer worker role encompassed a number of skills and abilities (described as qualities of the role) such as using intuition, taking initiative, belief in their ability to do the work, not giving up on people, and the innate ability to develop trust and relationships. Related studies have shown that peer workers are particularly adept at building trust and fostering positive change with clients, leveraging their experiential knowledge to create inclusive and impactful relationships [3, 53]. Providing insight into the most important qualities of peer work can be helpful in relation to identifying individuals who may become peer workers and supporting transitions into the role. There is a need for far greater organisational clarity regarding the peer worker role [54–56]. The qualities and key components outlined in this synthesis provide insights into how job descriptions can be crafted to enhance the clarity and contribution of peer worker roles. Such clarity can help to create a basis of addressing differences in approaches, and managing potential conflicts and tensions with other workers, as well as ensuring appropriate support is provided to those in these roles.

In all types of work in the substance use and homelessness sector, there are issues around setting boundaries [1, 48]. This is particularly acute for peer workers whose lived experiences are a key feature of their role and work with others. While sharing experiences was a way to connect, there is also a need for peer workers to set boundaries about what is shared, as well as creating boundaries related to work hours. The experiences of peer workers in this review, and related research, highlight the importance of giving back and being a role model for their communities. There appears to be a deep and enduring commitment to others and finding ways to manage this commitment, as well as care for oneself, is a unique situation for peer workers who are often members of the communities they serve. Other studies of peer work have also highlighted this, with the recognition that crises are not confined to working hours [7]. This highlights the importance of setting boundaries to support health and wellness for peer workers. However, boundary setting for peer workers, given their experiences, is not going to be the same as the guidelines for professionals who are situated differently. Notably, several studies talked about ‘consequences’ for crossing boundaries, without providing detail as to what these might be [48, 51]. Thus, there is a need for open and ongoing engagement around what constitutes safe boundaries in the context of peer work that supports and promotes the health and wellbeing of workers. Training around setting appropriate boundaries to keep peer workers safe and to support their wellbeing appears to be of particular importance.

As noted in this review, relationships with other peer workers are an important source of support, including opportunities to debrief. This peer-to-peer support, alongside other professional support and mentorship, may enhance role transitions and experiences [36, 57]. There are additional challenges that come from working in highly emotional and stressful situations and supporting individuals with complex needs including traumatic life experiences who may display challenging behaviours. Clinical supervision has been identified in four of the nine included studies as an important source of support to the peer workers. Our findings suggest that it is vital to distil and identify key components of adequate clinical supervision for those working at this intersection. Peer workers across the studies in this review highlighted the importance of positive factors such as the need for training, personal, professional and career development opportunities, as well as sustainable employment. For those who were in paid roles, adequate remuneration was highlighted as important. This concern is echoed by those working in multiple sectors (harm reduction services, mental health and alcohol and treatment services) [53, 58, 59]. Peer work is commonly considered ‘low barrier’ work (casual employment or volunteer) and experienced as ‘precarious’, due to lack of permanent employment that provides financial stability, benefits, and job security [58]. Given the diverse and extensive benefits of peer work that have been identified in this synthesis, there is an urgent need to recognise, formalise, properly remunerate, and provide additional training for these roles. The need for such professionalisation speaks to the organisational context and seeing peer workers as employees rather than volunteers. Clearly, transitioning to the role would be supported by clear job descriptions, stable working conditions, adequate pay and benefits alongside orientation and training [1].

There are key milestones in peer worker professional development, namely: orientation and training, adapting to organisational culture, managing relationships, and engaging with opportunities for professional development. A range of studies have identified a mix of formal training with on-the-job learning, yet detailed insights into peer worker experiences and evaluation of this process are limited [27, 50, 53, 57, 60–63]. In adapting to organisational culture, peer workers have faced challenges in integrating their experiential expertise within professional environments, navigating tensions between differently valued knowledge types (i.e., lived versus professional experience) [61, 64, 65]. However, the literature highlights a significant gap in the development of professional networks with other professionals in the field beyond organisational boundaries, where peer workers often feel undervalued by external non-peer colleagues. As reflected elsewhere, organisations employing peer workers across homelessness and substance use support have developed internal support systems to try to help manage and strengthen these external professional relationships [57, 60, 66, 67]. Lastly, opportunities for peer worker professional development remain under-explored in the literature, with only a limited number of studies indicating pathways to career advancement and a lack of exploration of transitions from volunteering to paid roles within organisations which is a common route for those with lived experience in this field [50].

In terms of implications, there is a clear evidence gap in understanding peer workers’ experiences of transitioning into such roles. This is important in terms of providing suitable support for those currently receiving support to subsequently become a peer worker, therefore building capacity in the field. Future research should explore these transitions. There is also limited research regarding the experiences of female peer workers in particular, who may be more likely to experience challenges of becoming peer workers and difficulties in their roles compared to males. Future research could explore these experiences in depth. There is a need for organisations employing peer workers to ensure they are fully integrated in teams, provided with appropriate training and development opportunities, stable working conditions, and supervision. The findings of this review provide insight into the factors that organisations may wish to consider when employing peer workers.

### Strengths and limitations

This qualitative evidence synthesis has provided insight into the experiences of peer workers providing support at the intersection of homelessness and substance use and is the first to synthesise these experiences. We ensured the review was conducted rigorously, by involving several authors at each stage of the process. The review was conducted by a wide interdisciplinary team with an international perspective, including those who have worked as peer workers. The findings of this synthesis, however, are based on the views of those only in the included studies and therefore represents a small sample of those working as peer workers with lived experience of homelessness and substance use. We specifically focused on studies that were conducted at this intersect, meaning studies that focused solely on substance use or homelessness were not included. Most of the studies were conducted in the UK (mainly England) which may limit transferability of findings to other settings, particularly in terms of differences between those currently using substances and those who may be abstinent, with peer workers’ current substance use reported only explicitly in North American studies [28, 53, 54]. As noted above, gender and ethnicity were not reported across all studies and therefore the findings are limited to the perspectives of a mostly male cohort of peer workers. Finally, the quality of the included studies, assessed using CASP, varied. Most studies were of moderate to high quality, and those with lower scores often did not contain sufficient detail regarding the quality appraisal components. Importantly, the majority reported rigorous data analysis. While some studies did not score highly using CASP, we had mainly low concerns about their methodological limitations. The themes presented in our review are supported by studies with methodological strengths and weaknesses, and for all themes the findings were discussed in at least six of the nine studies, ensuring that themes are based across a wide variety of studies and not just a few lower quality ones.

## Conclusion

In this qualitative evidence synthesis, we focused on peer work at the intersection of substance use and homelessness, revealing important insights for research, policy and practice related to peer worker role and qualities, benefits, and challenges as well as the importance of relationships. The role qualities highlight the value of lived experience as a way of creating supportive and trusting relationships with those they are working with. Relatedly, this created several challenges linked to the need for setting boundaries and managing relationships with other professionals who are situated differently with potentially different work responsibilities and roles despite sharing similar qualities. Other challenges related to organisational support such as training, career development and job security. From this review, we gleaned insights for those employing peer workers to support role transitions such as clear job descriptions that encompass peer worker qualities and the potential of peer-to-peer support in addition to other professional and organisational supports.

## Supplementary Information

Supplementary Material 1.

Supplementary Material 2.

## Data Availability

This study was a review of existing data, which is openly available at locations cited in the reference section. No new data were created or analysed in this study.

## Abbreviations

CASP: Critical Appraisal Skills Programme
ENTREQ: Enhancing transparency in reporting the synthesis of qualitative research
ETHOS: European typology of homelessness and housing exclusion
HIV: Human immunodeficiency virus
IPS: Intentional peer support
NHS: National Health Service
PRISMA: Preferred reporting items for systematic reviews and meta-analyses
PROSPERO: International prospective register of systematic reviews
RAP: Risk avoidance partnership
RCT: Randomised controlled trial
SPIDER: Sample, phenomenon of interest, design, evaluation, research type
TB: Tuberculosis
UK: United Kingdom
US: United States
