# Supplementary material for: “You are helping from the heart not just from the head”: a systematic review and qualitative evidence synthesis of the experiences of peer workers working with people experiencing homelessness and substance use
Source: BMC Public Health. 2025 May 9;25:1714. doi: 10.1186/s12889-025-23006-6 (PMC12063458; doi:10.1186/s12889-025-23006-6)
Supplement: Supplementary file 2 — Supplementary Material 2. [file 12889_2025_23006_MOESM2_ESM.docx]

Supplementary file 2. CASP quality appraisal

| **Source** | **Statement of aims** | **Appropriate method** | **Appropriate design** | **Appropriate recruitment** | **Appropriate data collection** | **Relationship between researcher and participant considered** | **Ethical issues considered** | **Rigorous data analysis** | **Statement of findings** | **How valuable is the research?** | **Total** |
| --- | --- | --- | --- | --- | --- | --- | --- | --- | --- | --- | --- |
| Annand et al.  (2022) | Yes | Yes | Yes | Yes | Yes | Not clear | Not clear | Not clear | Yes | Not clear | 6/10 |
| Barker et al. (2018) | Yes | Yes | Yes | Yes | Yes | Yes | Not clear | Yes | Yes | Yes | 9/10 |
| Croft et al. (2013) | Yes | Yes | Yes | Not clear | Not clear | Not clear | No | Not clear | Yes | Yes | 5/10 |
| MacLellan et al.  (2017) | Yes | Yes | Yes | Yes | Yes | Yes | Not clear | Yes | Yes | Yes | 9/10 |
| Parkes et al.  (2022) | Yes | Yes | Yes | Yes | Yes | No | Yes | Yes | Yes | Yes | 9/10 |
| Pauly et al. (2021) | Yes | Yes | Yes | Yes | Yes | Yes | Yes | Yes | Yes | Not clear | 9/10 |
| Surey et al. (2021) | Yes | Yes | Yes | Not clear | Yes | Yes | Not clear | Not clear | Yes | Yes | 7/10 |
| Tookey et al. (2018) | Yes | Yes | Yes | Yes | Yes | Yes | Yes | Yes | Yes | Yes | 10/10 |
| Weeks et al. (2006) | Not clear | Yes | Yes | Not clear | Not clear | No | Not clear | Yes | Not clear | Yes | 4/10 |
